# Effect of postpartum anaemia on maternal health-related quality of life: a systematic review and meta-analysis

**DOI:** 10.1186/s12889-022-12710-2

**Published:** 2022-02-21

**Authors:** Ernest Moya, Nomsa Phiri, Augustine T. Choko, Martin N. Mwangi, Kamija S. Phiri

**Affiliations:** 1 Training and Research Unit of Excellence (TRUE), 1 Kufa Road, Chichiri, BT3, PO Box 30538, Blantyre, Malawi; 2 Department of Public Health, School of Public Health and Family Medicine, College of Medicine, Kamuzu University of Health Sciences, Private Bag 360, Chichiri, BT3, Blantyre, Malawi; 3 College of Medicine, Malawi-Liverpool Wellcome Trust Queen Elizabeth Central Hospital, PO Box 30096, Chichiri, Blantyre, Malawi

**Keywords:** Anaemia, Iron deficiency anaemia, Depression, Fatigue, Mother–child interaction, Systematic review

## Abstract

**Background:**

Postpartum anaemia remains a persistent and severe public health issue in many parts of the world. Studies have reported mixed findings on the effects of anaemia during the postpartum period on maternal health-related quality of life (HRQoL). We conducted this systematic review to summarise available evidence to inform public health practitioners on whether 1) anaemia negatively impact maternal health-related quality of life and 2) whether iron supplementation in anaemic women can improve maternal HRQoL during the postpartum period.

**Methods:**

This review’s protocol was registered online with PROSPERO (CRD42020206618). We extensively searched Embase, PubMed, Cochrane and Scopus through the HINARI website to identify studies that reported either association or effect of postpartum anaemia on fatigue, depression and mother–child interaction. We restricted our search to studies of human females published in English language from databases inception until August 2020. We followed a Cochrane guideline for reporting systematic reviews and meta-analysis to synthesise data**.**

**Results:**

Twenty-seven studies were included in this systematic review, with some reporting all three domains (fatigue, depression and mother–child interaction) of HRQoL. Seven observational studies with pooled dichotomous outcomes showed that iron deficient or anaemic women were 1.66 times more likely to experience symptoms of depression than non-anaemic or iron-replete women [RR = 1.66 (95% CI: 1.28; 2.16), I^2^ = 67.0%, *P* < 0.01]. In three randomized controlled trials (RCTs), pooled continuous data showed statistically significant reduction in fatigue scores in women who received iron supplementation than the control group [MD: -1.85 (95% CI: -3.04; -0.67), I^2^ = 65.0%, *p* < 0.06]. Two of the four included studies showed that anaemic mothers were less responsive and had negative feelings towards their children than non-anaemic mothers.

**Conclusion:**

Evidence from this review suggests that postpartum anaemia negatively affects health-related quality of life and that iron replenishment improves both symptoms of fatigue and depression. Nevertheless, it remains unclear whether postpartum anaemia affects mother–child interaction.

**Supplementary Information:**

The online version contains supplementary material available at 10.1186/s12889-022-12710-2.

## Background

Postpartum anaemia remains a persistent and severe public health issue in many parts of the world [1]. The World Health Organisation defines postpartum anaemia as a haemoglobin concentration of < 11 g/dl at one week post-delivery and < 12 g/dl in the first postpartum year [2]. Although maternal iron stores are expected to replenish after delivery, the prevalence of anaemia in women after childbirth remains unacceptably high in both developed (22–50%) and developing (50–80%) countries [3]. Postpartum anaemia is mainly caused by untreated antenatal iron deficiency or anaemia and excessive blood loss during or after childbirth [3]. Postpartum anaemia is classified as anaemia due to iron deficiency in many women [1, 4]. Iron deficiency anaemia (IDA) is the state in which there is insufficient body iron to maintain the tissue’s normal physiological function, i.e. blood, brain and muscles [5]. A reduction of serum ferritin below 30 µg/l in settings where inflammatory conditions are uncommon is suggestive of iron deficiency (ID) [5].

Untreated postpartum anaemia affects the wellbeing of both the mother and child. Maternal ID or anaemia related complications may impair physical capacity and performance and negatively impact health-related quality of life [6]. Health-Related Quality of Life (HRQoL) is the patient’s self-report on how her wellbeing and functioning level are affected by individual health or medical treatment received [7]. There is now consensus that HRQoL is a multidimensional construct. At a minimum, it consists of the physical, mental and social domains. Symptoms such as fatigue, psychological distress (anxiety and depression) and altered mother–child relationship, among others, are common indicators of poor HRQoL.

Since health care is becoming more patient-centred, patient-reported outcomes such as quality of life are increasingly important [8]. Worldwide, studies have reported mixed results on the association between postpartum anaemia and maternal HRQoL. For example, Chandrasekaran et al. (2018) [9] reported no association between postpartum anaemia and HRQoL. Conversely, Khalafallah et al. (2012) [10] found a strong association between maternal iron stores and improved HRQoL. Due to controversies surrounding the role of iron deficiency or anaemia on maternal HRQoL and the fact that improved maternal HRQoL after childbirth is linked to the improved wellbeing of the child and “family and society”[11]; reaching a consensus can be influential in deciding whether postpartum iron supplementation improves maternal wellbeing. Therefore, we conducted a systematic review to determine the effect of postpartum anaemia on maternal HRQoL in the first postpartum year. In assessing the effect of iron deficiency or anaemia on HRQoL, we used the Wilson and Cleary model [12], which links the biological and physiological variables (iron and haemoglobin levels) to measures of HRQoL such as symptom status (fatigue, depression) and functional status (mother–child interaction).

## Method

### Protocol registration

This review’s protocol was registered online with PROSPERO (CRD42020206618) following the Preferred Reporting Items for Systematic Reviews and Meta-analysis [13].

### Search Strategy

We searched PubMed, Embase, Cochrane and Scopus through the HINARI website. For PubMed, we used English MeSH keywords: “anaemia”, “iron deficiency”, “health-related quality of life”, “depression”, “bonding” and “postpartum women” with attention to possible synonyms, spelling variants, and correct use of truncation and Boolean operators (Additional file 1). Using search terms for PubMed, we developed a search strategy for Embase by entering one term at a time, and a correct term was selected on Emtree (Additional file 1). We restricted our search to studies of human females published in English from databases inception until August 2020. After that, the results were directly exported into EndNote reference management software (Endnote 2017), and all duplicates were removed. We also manually searched references of included articles for additional relevant studies.

### Study Selection (inclusion and exclusion)

Studies were included according to PICO: 1) Population: epidemiological studies (randomised and non-randomised-controlled trials, cohort, case–control and longitudinal cross-sectional studies) that reported either the effect or association between anaemia or iron deficiency during the postpartum period [14] and measure of HRQoL either fatigue or depression or mother–child interaction; 2) Intervention/Exposure: any form of postpartum iron supplementation or haematological test confirming anaemia/iron deficiency and questionnaire confirming PPD or fatigue or mother–child interaction; 3) **C**omparison: placebo or standard treatment or those that showed HRQoL indicators in anaemic and non-anaemic women and 4) Outcome; effect of (experimental studies) or association (observational studies) of anaemia or iron deficiency on fatigue, depression and mother–child interaction. Other study designs such as cross-sectional, case series, narrative reviews and commentaries were excluded. Additionally, qualitative study designs were excluded from this review.

### Data extraction and management

A standardised, piloted data extraction form was used to extract data from included studies. Two independent reviewers (EM and NP), working in parallel, screened titles and abstracts of the identified articles. After that, full articles were retrieved for further evaluation. Discrepancies between the two reviewers were resolved through discussion. Disagreements between the two were resolved through discussion with MNM and KP. For studies with dichotomous outcomes, we extracted the number of events and participants in each group. We extracted the effect measure, which included both crude and adjusted ratios with their respective 95% confidence intervals and p-values. We extracted means and standard deviation for continuous outcome with normally distributed data while medians, range and p-value of the non-parametric test were extracted for continuous skewed data. We also extracted correlation coefficients for correlation studies and median or mean change from baseline for longitudinal studies.

### Risk of bias assessment

Two reviewers (EM and NP) independently assessed the risk of bias in the included studies, and discussions resolved disagreements. For randomised trials, we used a revised Cochrane risk-of-bias tool for randomised trials (RoB-2). Thereafter the risk of bias in the individual study was judged as either “*low risk*” or “*moderate risk*” or “*high-risk bias*” [14]. The Newcastle–Ottawa Scale was used to assess the risk of bias for cohort studies and case–control studies [15]. We considered studies rated with ≥ 7 stars as good (moderate risk) [16]. We adopted and modified a tool for evaluating the risk of bias in non-randomised studies of interventions (ROBINS-I) for longitudinal observational studies [17]. We assessed bias due to confounding, selection of participants, missing data, measurement of outcome, and selecting the reported outcomes. We dropped two domains that assess bias due to intervention classification and deviations from the intended interventions as these were deemed not applicable [17].

### Grading of evidence

We graded the quality of evidence using the Grading of Recommendations Assessment, Development and Evaluation (GRADE) [18]. The assessment criteria included; risk of bias, indirectness, imprecision, inconsistency and publication bias. Thereafter, the strength of evidence was grouped as “high”, “moderate”, “low”, or “very low”.

### Data synthesis

Data analysis was performed using Stata SE for Windows V.14.1 (StataCorp, College Station, Texas, USA). Data has been grouped and analysed separately depending on study design (observation and experimental) and whether the study has reported the effect of maternal anaemia on either fatigue, depression or mother–child interaction. We presented risk ratios (RR) for study level comparison of binary outcomes and mean difference (MD) for the pooled continuous data. We used Mantel–Haenszel random-effects model to calculate pooled RR and 95% confidence interval (CI). A random-effects model was used to calculate pooled MDs. We used recommended formulas to calculate the estimated sample mean and SD from the sample size, median, range and/or interquartile ranges [19]. Wherever fatigue was reported with multiple scales, such as on the multi-dimension fatigue inventory (MFI) scale, the overall “general fatigue” was selected. The I^2^ statistic was used to quantify statistical heterogeneity. We used Cochrane recommendation to interpret I^2^ statistics as “might not be important” (0—40%), “moderate” (30 – 60%), “substantial” (50—90%) or “considerable” (75%—100%). We planned to assess publication bias using funnel plots, but this was not possible because, in each meta-analysis, there were less than ten studies. Thus, we used visual inspection of the confidence intervals. The findings were considered to be statistically significant if the reported P-value was < 0.05.

## Results

### Study characteristics and methodological quality

**Fig. 1:**
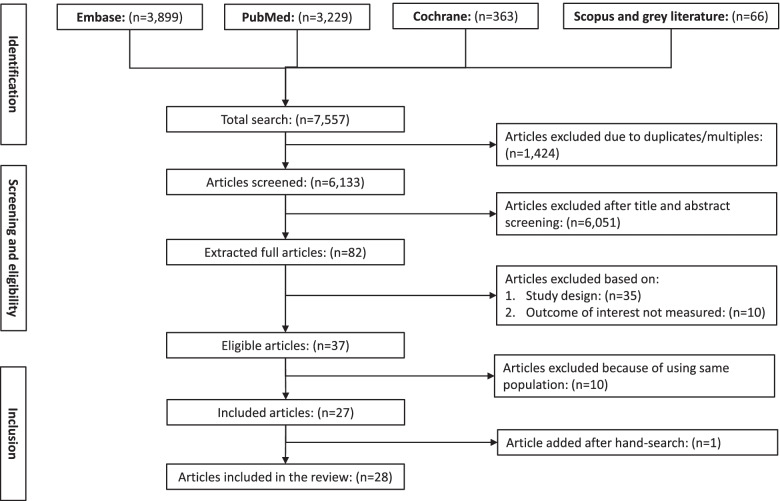
Process of selecting studies for inclusion in the systematic review

The searches in Embase, PubMed, Cochrane Central Trial, and Scopus databases through the Hinari website identified 7,547 citations, of which 82 (1.1%) full articles were extracted and assessed for their eligibility. Of the 82 articles, 27 (32.9%) met the eligibility criteria. We further included one article identified by searching the references of the included articles (Fig. 1). Tables 1, 2 and 3 summarise the characteristics and methodological quality of the eligible studies.

**Table 1:** Study characteristics and association between anaemia or iron deficiency during postnatal period and maternal postpartum depression

Author & Year	Study Design and Setting	Risk of Bias	Sample Size	Assessment Tool	Effect measure and Significance
Sheikh et al. 2015 [20]	RCT double-blind, placebo; Iran	Low	*N* = 70 (iron supplementation; 35, placebo; 35)	EPDS (cut off: ≥ 11)	*p* = 0.03
Perello et al. 2014 [21]	RCT double-blind; Spain	Low	*N* = 72 anaemic (ferrous sucrose + oral iron; 36, placebo + oral iron; 36)	EPDS (cut off: ≥ 11)	*P* > 0.05
Beard et al. 2005 [22]	RCT, double-blind; South Africa	Low	*N* = 95 (30 in IDA-PL, 34 in IDA-Ferrous, & 31 in control)	EPDS (cut off: ≥ 10)	*P* < 0.005
Hamm et al. 2020 [23]	RCT open label; USA	Moderate	*N* = 66 (single RBCs; 33, multiple RBCs; 33)	EPDS (mean scores)	4 (1–11) vs 5.5 (2–8) *p* = .34
Holm et al. 2019 [24]	RCT open-label; Denmark	Moderate	*N* = 85 (Oral iron; 44, and Iron isomaltoside; 41)	EPDS (mean scores)	MD: -2.39 (95% CI: -3.62; -1.16) (*p* = .0002)
Paoletti et al. 2013 [25]	RCT open-label; Italy	Moderate	*N* = 852 (Group A: 424, Group B: 428)	EPDS (cut off: ≥ 12)	*P* < 0.05
Maeda et al. 2019 [26]	Prospective cohort; Japan	Moderate	*N* = 1128	EPDS ( cut off; ≥ 9)	AOR: 1.63 (95% CI: 1.17; 2.26)
Chandrasekaran et al. 2018 [9]	Prospective Observational; Canada	Moderate	*N* = 145/248	EPDS ( cut off; ≥ 10)	OR: 0.69 (95% CI: 0.15; 2.49)
Eckerdal et al. 2016 [27]	Nested Cohort; Sweden	Moderate	*N* = 446	EPDS (cut off; ≥ 12)	OR: 2.29 (95% CI:1.15; 4.58)
Alharbi et al. 2014 [28]	Case–Control; Saudi Arabia	Moderate	*N* = 352 (Case: 117, Control: 235)	EPDS ( cut off; ≥ 10)	AOR: 1.70 (95% CI: 1.05; 2.74) *P* = 0.03
Goshtasebi et al. 2013 [29]	Prospective Observational; Iran	Moderate	*N* = 281	EPDS (cut off ≥ 13)	AOR: 4.64 (95% CI: 1.33; 16.08)
Armony-Sivan et al. 2012 [30]	Prospective Observational; China	Moderate	*N* = 248 (confirmatory study)	EPDS ( cut off; ≥ 10)	*r* = 0.07
Albacar et al. 2010 [31]	Prospective Cohort; Spain	Moderate	*N* = 729	EPDS ( cut off; ≥ 9)	OR: 3.73 (95% CI: 1.84; 7.56) *P* = 0.0001
Miller et al. 2016 [32]	Prospective Observational; USA	High	*N* = 63	EPDS (cut off; ≥ 10)	*P* > 0.05
Corwin et al. 2003 [33]	Prospective Observational; USA	High	*N* = 37	CES-D	*r* = -0.381, *P* = 0.020
Paterson et al. 1994 [34]	Prospective observational; United Kingdom	High	*N* = 1010	EPDS scores	*P* > 0.05

***Abbreviations*:** *AOR* Adjusted Odds ratio, *CES-D* Epidemiological Studies-Depressive Symptomatology Scale, *EPDS* Edinburg Postpartum Depression Scale, *IDA-PL* Iron deficiency anaemia-placebo, *r* Pearson correlation coefficient

**Table 2:** Study characteristics and the association between anaemia or iron deficiency during postnatal period and maternal fatigue

Author & Year	Study Design and Setting	Risk of Bias	Sample Size	Assessment Tool	Significance
Hamm et al. 2020 [23]	RCT open-label; USA	Moderate	*N* = 66 (single RBCs: 33, multiple RBCs; 33)	MFI	*P* = 0.13
Holm et al. 2019 [24]	RCT open-label; Denmark	Moderate	*N* = 85 (Oral iron; 44, and Iron isomaltoside; 41)	MFI	*P* < .0001
Prick et al. 2014 [35]	RCT open-label; Netherlands	Moderate	*N* = 521 (Non-intervention; 262, RBCs; 259)	MFI and SF-36	*P* = 0.01
Westad et al. 2008 [36]	RCT open-label; Norway	Moderate	*N* = 128 (IV + oral iron; 58, oral iron only; 70	MFI and SF-36	*P* = 0.03
Hatzis et al. 2003 [37]	Matched intervention trial; Greece	Moderate	*N* = 74 (EPO; 37 and oral iron; 37)	Physical assessment: clinical symptoms of fatigue	*P* = 0.0012
Chandrasekaran et al. 2018 [9]	Prospective Observational; Canada	Moderate	*N* = 248	SF-36	OR: 1.03 (95% CI: 0.34; 2.94)
Van Der Woude et al. 2014 [8]	Prospective Cohort; Netherlands	Moderate	*N* = 220 (Anaemic; 112, No anaemia; 108)	SF-36	*P* = 0.008^a^
Miller et al. 2016 [32]	Prospective Observational; USA	High	*N* = 63	SF-36 and MFI	*P* > 0.05
Jansen et al. 2007 [38]	Prospective Cohort: Netherlands	High	*N* = 141	MFI	*P* = 0.002
Lee et al. 1999 [39]	Prospective Observational; USA	High	*N* = 30	Lee Fatigue Scale	*r* = -.27, *p* < .05^b^ *r* = -.44, *p* = .01^c^
Paterson et al. 1994 [34]	Prospective Observational; United Kingdom	High	*N* = 1010	Physical assessment: feeling low energy	*P* = 0.05

***Abbreviations***: *IV* intravenous *MFI* multi-fatigue dimension inventory, *SF-36* Short Form-36, *RCT* randomised controlled trial, *RBCs* Red Blood Cells, *r* Pearson correlation coefficient

^a^Un-adjusted

^b^Low ferritin levels

^c^Low haemoglobin levels

**Table 3:** Study characteristics and association between anaemia or iron deficiency during postnatal period and mother–child interaction or bonding

Author & Year	Study Design and Setting	Risk of Bias	Sample Size	Assessment Tool	Effect Measure and Significance
Murray-Kolb et al. 2009 [40]	RCT double-blind, placebo; South Africa	Low	*N* = 95 (IDA-PL; 30, IDA-Fe; 34, and control; 31)	Video recorder	*P* = 0.007–0.032^a^
Perez et al. 2005 [41]	RCT double-blind; South Africa	Low	*N* = 81 (IDA-PL; 21, IDA-Fe; 30 and Control; 30)	Parent/Caregiver Involvement Scale	*P* < 0.05
Hamm et al. 2020 [23]	RCT open-label; USA	Moderate	*N* = 66 (single RBCs; 33, multiple RBCs; 33)	Maternal Attachment Inventory	104 (102–104) vs 104 (102–104) *p* = 0.55^b^
Dearman et al. 2012 [42]	Case–Control; England	Moderate	*N* = 115 (anaemic; 57, non-anaemic; 58)	Postpartum Bonding Questionnaire	*P* > 0.05

**Key:** ^a^Ranges in subscale analysis; maternal sensitivity (*p* = 0.032), structuring (*p* = 0.026), and non-hostility (*p* = 0.007)

^b^median score (range)

### Postpartum anaemia and maternal depression

Out of 18 studies that reported the effect or association between postpartum anaemia and depression, 15 studies were planned to be included in a meta-analysis. Meyer et al. (1995) [43] was not included as the study reported symptoms of postpartum blues and not depression. Holm et al. (2019) [24] and Güven et al. (2020) [44] were excluded due to a lack of sufficient information. Authors for both studies were contacted but did not provide the missing information by the time submission was made. However, all the three excluded studies reported a significant decrease in depression scores with a corresponding increase in haematological parameters (Table 1).

**Fig. 2:**
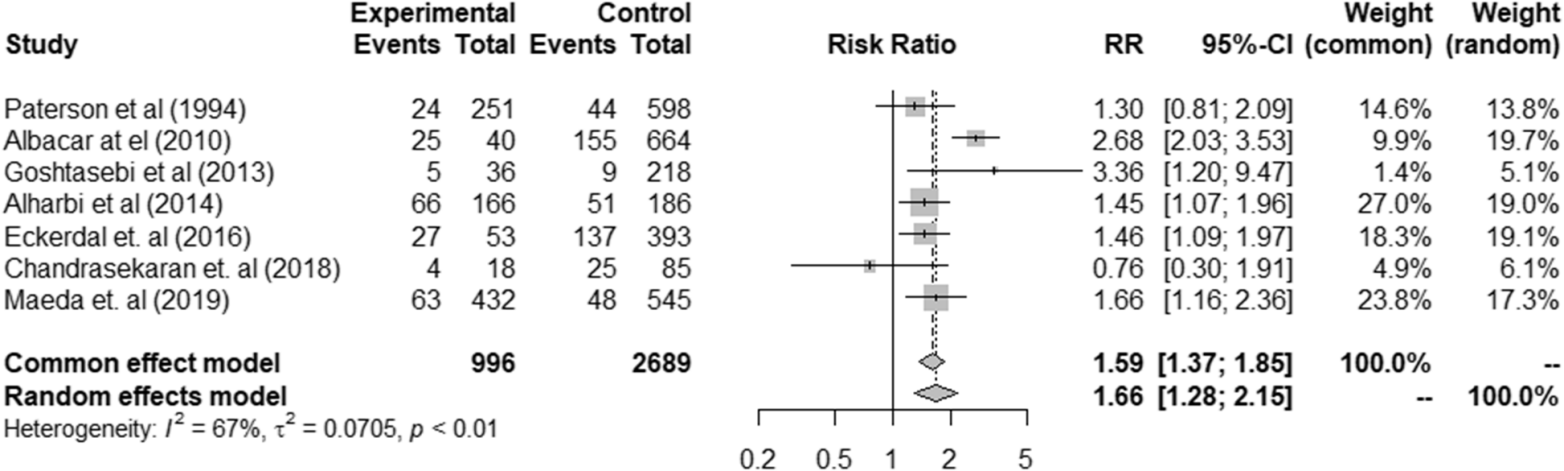
Forest plot of observational studies showing the association of anaemia during the postnatal period and maternal postpartum depression

Ten of the remaining 15 studies were observational studies [9, 21, 25–32] and 5 were RCTs. [20, 22, 23, 33, 34] The comparison of depression scores in eight observational studies was dichotomous. One study [32] was not included in a meta-analysis as it was not consistent with zero events in the non-exposed group. The pooled results of the remained seven studies [21, 25–32] showed that iron deficiency or anaemic women were 1.66 times more likely to experience symptoms of depression than the non-anaemic or iron-replete women and the findings were statistically significant [(heterogeneity estimate: I^2^ = 67.0%, *p* < 0.01), and RR = 1.66 (95% CI: 1.28; 2.15)] (Fig. 2). Two observational studies [29, 31] measured depression as a continuous outcome, and their findings were not pooled together but reported separately (Table 1).

**Fig. 3:**
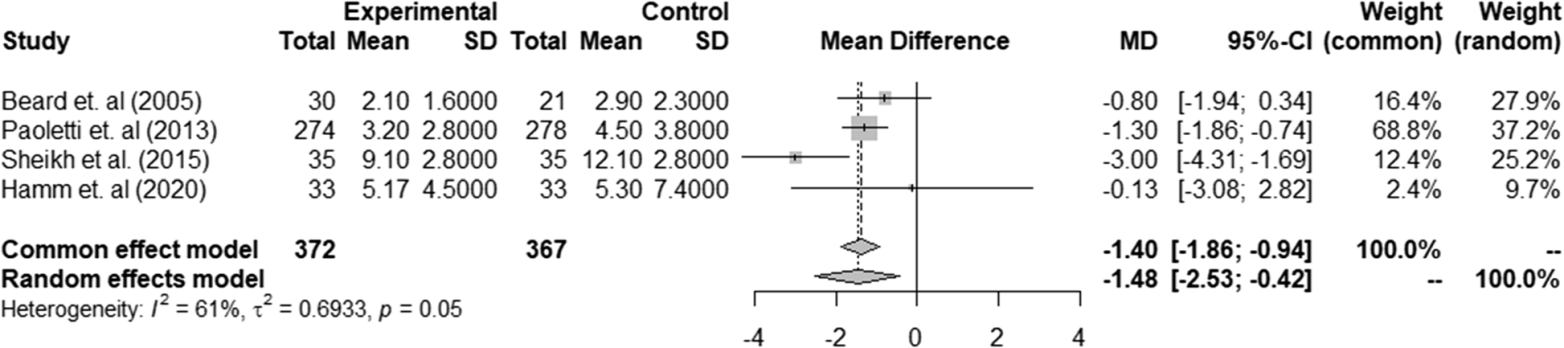
Forest plot of Randomised Control Trials on the effect of iron supplementation during the postnatal period on maternal postpartum depression

Similar findings were observed in four of five RCTs [20, 22, 23, 25] that measured depression as a continuous outcome. The pooled results in these four studies [*n* = 739 (treatment group = 372 and control group = 367)] showed a significant reduction in depression scores for women who were given intravenous iron than oral iron or placebo group based on random mixed model (MD = -1.48, 95% CI: -2.53; -0.42). However, heterogeneity was considerable high (I^2^ = 61.0%, *p* = 0.05) (Fig. 3). Perello et al. (2014) [21] was not included in the meta-analysis as the outcome measure depression was reported differently with others ( categorical outcome) and findings in this study are present in Table 1.

### Postpartum anaemia and fatigue

Eleven studies reported either the effect or association between postpartum anaemia and fatigue (Table 2). Four studies [27, 32, 38, 39] were excluded from a meta-analysis due to insufficient information. Westad et al. (2008) [36] reported marked improvement in physical fatigue scores corresponding with rapid iron stores replenishment in women in the intervention group than the control group at 4, 8 and 12 weeks postpartum (*p* = 0.02, *p* = 0.02 and *p* = 0.03 respectively). However, it was excluded from analysis as the author did not provide missing information until submission.

**Fig. 4:**
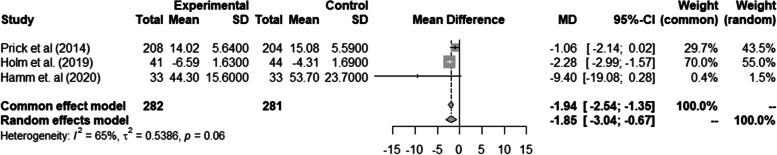
Forest plot of Randomised Control Trials on the effect of supplementation during the postnatal period on maternal postpartum fatigue

Pooled continuous data from three RCTs [20, 22, 24] (Fig. 4) showed statistically significant reduction of fatigue scores in women who received iron supplementation or vitamins that led to haematological increase than the control (MD: -1.85, 95% CI: -3.04; -0.67) but with considerable heterogeneity (I^2^ = 65.0%, *p* < 0.06). Fatigue scores were reported as a categorical outcome in two studies. Hatzis et al. (2003), [37] in their matched intervention study in Greece, reported that women who received recombinant human erythropoietin reported fewer clinical symptoms of fatigue (*p* = 0.0012) compared to oral iron. Conversely, Chandrasekaran et al. (2018) [9] reported no significant association between anaemia and maternal functional status (OR: 1.03, 95% CI: 0.34, 2.94) (Table 2).

### Postpartum anaemia and mother–child interaction

Four studies reported the effect of postpartum anaemia on mother–child interaction (Table 3). Murray-Kolb et al. (2009) [40] reported that at nine months’ post-intervention, women who were not anaemic and the IDA-ferrous groups significantly improved scored significantly better on maternal sensitivity, non-hostility, and structuring scales and child responsiveness scale than did the IDA-placebo group (p-value = 0.007), whose iron stores remained low. Similarly, Perez et al. (2005) [41] reported that anaemic mothers in the IDA-placebo group had negative statements towards their infants, less goal setting and responsiveness than mothers in non-anaemic and IDA-ferrous groups (*p* < 0.05) at nine months postpartum.

Unlike the above findings, Hamm et al. (2020) [23] reported that significant improvement in Hb levels in women who received multiple units of RBCs (8.7 g/dl versus 7.8 g/dl) did not significantly improve maternal attachment scores. Dearman et al. (2012) [42], in their pilot case–control study enrolled 115 women (Hb < 10.5 g/dl = 57 and non-anaemic = 58) and reported no statistical difference in maternal perception of mother-infant bonding between the anaemic and non-anaemic group.

### Quality of evidence

We used the GRADE approach to judge the strength of the evidence. Table 4 has provided the GRADE summaries with an overall quality evaluated as “moderate”. The risk of bias was judged to be moderate, and inconsistency was assessed using the heterogeneity statistics.

**Table 4:** GRADE assessment of confidence in effect size

Outcome	Risk of Bias	Consistency	Indirectness	Imprecision	Publication Bias	Strength of Evidence
Postpartum anaemia or ID on PPD (Observational Studies)	No serious limitation	No serious limitation	No serious limitation	No serious limitation	No serious limitation	Moderate
Postpartum anaemia or ID on PPD (RCTs)	No serious limitation	No serious limitation	No serious limitation	No serious limitation	No serious limitation	Moderate
Postpartum anaemia or ID on fatigue (continuous data)	No serious limitation	No serious limitation	No serious limitation	No serious limitation	No serious limitation	Moderate

*GRADE* Grading of Recommendations Assessment, Development and Evaluation

*RCTs* Randomised Control Trials

## Discussion of results

The purpose of the present study was to determine the effect of postpartum anaemia on the three domains of HRQoL in a postpartum woman. Studies have reported mixed findings on the association between iron deficiency and postpartum depression. This is despite the physiological link that iron is required to produce the behavioural neurotransmitter dopamine, and lack of it is clinically manifested by symptoms such as depression and fatigue [45]. Our pooled results from comparisons of dichotomous data from observational studies indicated that iron deficiency or anaemia measured at different time points during the first postpartum year is a significant risk factor for postpartum depression. Our findings are similar to those of a recent meta-analysis which also reported significant associations between postpartum anaemia and symptoms of depression [46].

The other important findings from the pooled continuous data from RCTs showed that iron supplementation significantly increased iron and haemoglobin levels with a corresponding significant decrease in depression scores. The heterogeneity test (I^2^ = 61%) was substantially high with a borderline significant value (*p* = 0.05). Variations in the type of the interventions and comparators (such as intravenous iron versus oral iron/placebo, a single unit of blood versus multiple units), use of different intervention dosages and variations in the timing of measuring depression might explain the observed substantial heterogeneity.

Two important findings on the effect of postpartum anaemia on maternal fatigue were noted. Firstly, pooled evidence suggests that anaemia as indicated by low Hb or depleted iron stores is associated with maternal postpartum fatigue, and can be treated by iron replenishment [I^2^ = 65.0%, *p* = 0.06, (MD: -1.85, 95% CI: 3.04; -0.67)] in either strategy. This finding is similar to a previous systematic review investigating the impact of intravenous iron treatment on HRQoL in patients with IDA [47]. Secondly, little attention has been paid to the same condition in developing countries, and it remains unclear whether the above findings are applicable in the African context. A meta-analysis by Badr et al. (2017) [48] aimed at identifying predicting factors for maternal postpartum fatigue called authors to explore whether race/geographical region can mediate the association between iron deficiency and postpartum fatigue.

The association between anaemia and mother–child interaction/bonding remains unclear. Globally, researchers have paid little attention to this area. We only identified four studies that showed mixed findings, and their conclusions were not pooled due to the outcome being reported differently among studies. Two well-conducted studies [40, 41] reported that maternal anaemia negatively impacted mother–child interaction. Other studies that reported no association also had some shortfalls. For example, the sample size in Hamm et al. (2020) [23] was not powered to evaluate the effects of anaemia on secondary outcomes such as mother–child attachment. Dearman et al. (2012) [42] also acknowledged that lack of association in their study might be due to the small sample size lacking power.

### Study Limitation

Our study has two major limitations. Firstly, we limited our search to human studies published in English language only because none of the authors is conversant with other languages. Secondly, we did not produce funnel plots to assess publication bias in the included studies, as less than ten studies were included in each meta-analysis.

## Conclusion

To our knowledge, this is the first systematic review that has determined the effect of postpartum anaemia on maternal health-related quality of life in a holistic approach by incorporating all domains of HRQoL. While it is clear from our findings that postpartum anaemia negatively affects health-related quality of life domains of physical and mental health and that iron replenishment tremendously improved symptoms of fatigue and depression, it remains unclear on its impact on mother–child interaction. There is a paucity of data from developing countries on the effect of postpartum anaemia on HRQoL. We, therefore, call for well-designed studies in Africa to provide contextual evidence. Nonetheless, we agree and call upon clinicians in developing countries to adhere to the World Health Organisation recommendation of routine iron supplementation to women until six weeks postpartum to improve maternal HRQoL during the postpartum period.

## Supplementary Information

**Additional file 1: **Search Strategies.

## Data Availability

We used data from previously published articles that have been cited in this published article. Other resources which includes data extraction forms, and detailed information pertaining risk of bias can be provided by corresponding author upon request.

## Abbreviations

CES-D: Center for Epidemiological Studies Depression Scale
EPDS: Edinburgh Postpartum Depression Scale
HRQoL: Health-Related Quality of Life
IDA: Iron Deficiency Anaemia
MFI: Multidimensional Fatigue Inventory scale
RBCs: Red Blood Cells
SF-36: 36-Item Short Form Survey
RCTs: Randomised Control Trials
