# Supplementary material for: Effect of postpartum anaemia on maternal health-related quality of life: a systematic review and meta-analysis
Source: BMC Public Health. 2022 Feb 21;22:364. doi: 10.1186/s12889-022-12710-2 (PMC8862508; doi:10.1186/s12889-022-12710-2)
Supplement: Supplementary file 1 — Additional file 1: Search Strategies. [file 12889_2022_12710_MOESM1_ESM.docx]

**Appendix 1: Search Strategies**

**Database: PubMed <Hinari Access to Research for Health programme> 2020 August 14**

1. ((((((((anemia) OR (anaemia)) OR (hemoglobin)) OR (haemoglobin)) OR (Hb)) OR (ferritin)) OR (iron deficiency)) OR (soluble transferrin receptor)) OR (IDA)
2. ((((((((((((((((health related quality of life) OR (HRQoL)) OR (health status)) OR (fatigue)) OR (tiredness)) OR (weariness)) OR (feeling worn out)) OR (physical functioning)) OR (role limitation)) OR (bodily pain)) OR (general health)) OR (wellbeing)) OR (vitality)) OR (social functioning)) OR (cognitive functioning)) OR (depression)) OR (anxiety)
3. (((((postpartum women) OR (postnatal women)) OR (breastfeeding women)) OR (lactating mothers)) OR (puerperal)) OR (maternal)
4. 1 AND 2 AND 3. ((((((((((anemia) OR (anaemia)) OR (hemoglobin)) OR (haemoglobin)) OR (Hb)) OR (ferritin)) OR (iron deficiency)) OR (soluble transferrin receptor)) OR (IDA)) AND (((((((((((((((((health related quality of life) OR (HRQoL)) OR (health status)) OR (fatigue)) OR (tiredness)) OR (weariness)) OR (feeling worn out)) OR (physical functioning)) OR (role limitation)) OR (bodily pain)) OR (general health)) OR (wellbeing)) OR (vitality)) OR (social functioning)) OR (cognitive functioning)) OR (depression)) OR (anxiety))) AND ((((((postpartum women) OR (postnatal women)) OR (breastfeeding women)) OR (lactating mothers)) OR (puerperal)) OR (maternal))

("anaemia"[All Fields] OR "anemia"[MeSH Terms] OR "anemia"[All Fields] OR "anaemias"[All Fields] OR "anemias"[All Fields] OR ("anaemia"[All Fields] OR "anemia"[MeSH Terms] OR "anemia"[All Fields] OR "anaemias"[All Fields] OR "anemias"[All Fields]) OR ("haemoglobin"[All Fields] OR "hemoglobins"[MeSH Terms] OR "hemoglobins"[All Fields] OR "hemoglobin"[All Fields] OR "haemoglobins"[All Fields] OR "hemoglobin s"[All Fields] OR "hemoglobine"[All Fields] OR "hemoglobinization"[All Fields] OR "hemoglobinized"[All Fields]) OR ("haemoglobin"[All Fields] OR "hemoglobins"[MeSH Terms] OR "hemoglobins"[All Fields] OR "hemoglobin"[All Fields] OR "haemoglobins"[All Fields] OR "hemoglobin s"[All Fields] OR "hemoglobine"[All Fields] OR "hemoglobinization"[All Fields] OR "hemoglobinized"[All Fields]) OR "Hb"[All Fields] OR ("ferritin s"[All Fields] OR "ferritine"[All Fields] OR "ferritins"[MeSH Terms] OR "ferritins"[All Fields] OR "ferritin"[All Fields]) OR (("iron"[MeSH Terms] OR "iron"[All Fields]) AND ("deficiences"[All Fields] OR "deficiencies"[All Fields] OR "deficiency"[MeSH Subheading] OR "deficiency"[All Fields] OR "deficient"[All Fields] OR "deficients"[All Fields])) OR (("solubility"[MeSH Terms] OR "solubility"[All Fields] OR "solubilities"[All Fields] OR "soluble"[All Fields] OR "solubles"[All Fields] OR "solublization"[All Fields] OR "solublize"[All Fields] OR "solublized"[All Fields]) AND ("receptors, transferrin"[MeSH Terms] OR ("receptors"[All Fields] AND "transferrin"[All Fields]) OR "transferrin receptors"[All Fields] OR ("transferrin"[All Fields] AND "receptor"[All Fields]) OR "transferrin receptor"[All Fields])) OR "IDA"[All Fields]) AND ("quality of life"[MeSH Terms] OR ("quality"[All Fields] AND "life"[All Fields]) OR "quality of life"[All Fields] OR ("health"[All Fields] AND "related"[All Fields] AND "quality"[All Fields] AND "life"[All Fields]) OR "health related quality of life"[All Fields] OR ("hrqols"[All Fields] OR "quality of life"[MeSH Terms] OR ("quality"[All Fields] AND "life"[All Fields]) OR "quality of life"[All Fields] OR "hrqol"[All Fields]) OR ("health status"[MeSH Terms] OR ("health"[All Fields] AND "status"[All Fields]) OR "health status"[All Fields]) OR ("fatiguability"[All Fields] OR "fatiguable"[All Fields] OR "fatigue"[MeSH Terms] OR "fatigue"[All Fields] OR "fatigued"[All Fields] OR "fatigues"[All Fields] OR "fatiguing"[All Fields] OR "fatigueability"[All Fields]) OR ("fatigue"[MeSH Terms] OR "fatigue"[All Fields] OR "tiredness"[All Fields]) OR ("fatigue"[MeSH Terms] OR "fatigue"[All Fields] OR "weariness"[All Fields] OR "weary"[All Fields]) OR (("emotions"[MeSH Terms] OR "emotions"[All Fields] OR "feeling"[All Fields] OR "feelings"[All Fields] OR "feels"[All Fields]) AND "worn"[All Fields] AND "out"[All Fields]) OR (("physical examination"[MeSH Terms] OR ("physical"[All Fields] AND "examination"[All Fields]) OR "physical examination"[All Fields] OR "physical"[All Fields] OR "physically"[All Fields] OR "physicals"[All Fields]) AND ("functional"[All Fields] OR "functional s"[All Fields] OR "functionalities"[All Fields] OR "functionality"[All Fields] OR "functionalization"[All Fields] OR "functionalizations"[All Fields] OR "functionalize"[All Fields] OR "functionalized"[All Fields] OR "functionalizes"[All Fields] OR "functionalizing"[All Fields] OR "functionally"[All Fields] OR "functionals"[All Fields] OR "functioned"[All Fields] OR "functioning"[All Fields] OR "functionings"[All Fields] OR "functions"[All Fields] OR "physiology"[MeSH Subheading] OR "physiology"[All Fields] OR "function"[All Fields] OR "physiology"[MeSH Terms])) OR (("role"[MeSH Terms] OR "role"[All Fields]) AND ("limit"[All Fields] OR "limitation"[All Fields] OR "limitations"[All Fields] OR "limited"[All Fields] OR "limiting"[All Fields] OR "limits"[All Fields])) OR ("bodily"[All Fields] AND ("pain"[MeSH Terms] OR "pain"[All Fields])) OR (("drugs, generic"[MeSH Terms] OR ("drugs"[All Fields] AND "generic"[All Fields]) OR "generic drugs"[All Fields] OR "generic"[All Fields] OR "family characteristics"[MeSH Terms] OR ("family"[All Fields] AND "characteristics"[All Fields]) OR "family characteristics"[All Fields] OR "generation"[All Fields] OR "generations"[All Fields] OR "general"[All Fields] OR "general s"[All Fields] OR "generalisability"[All Fields] OR "generalisable"[All Fields] OR "generalisation"[All Fields] OR "generalization, psychological"[MeSH Terms] OR ("generalization"[All Fields] AND "psychological"[All Fields]) OR "psychological generalization"[All Fields] OR "generalization"[All Fields] OR "generalisations"[All Fields] OR "generalise"[All Fields] OR "generalised"[All Fields] OR "generalises"[All Fields] OR "generalisibility"[All Fields] OR "generalising"[All Fields] OR "generalities"[All Fields] OR "generality"[All Fields] OR "generalizability"[All Fields] OR "generalizable"[All Fields] OR "generalizations"[All Fields] OR "generalize"[All Fields] OR "generalized"[All Fields] OR "generalizes"[All Fields] OR "generalizing"[All Fields] OR "generally"[All Fields] OR "generals"[All Fields] OR "generate"[All Fields] OR "generated"[All Fields] OR "generates"[All Fields] OR "generating"[All Fields] OR "generation s"[All Fields] OR "generational"[All Fields] OR "generative"[All Fields] OR "generatively"[All Fields] OR "generativity"[All Fields] OR "generator"[All Fields] OR "generator s"[All Fields] OR "generators"[All Fields] OR "generically"[All Fields] OR "genericity"[All Fields] OR "generics"[All Fields]) AND ("health"[MeSH Terms] OR "health"[All Fields] OR "health s"[All Fields] OR "healthful"[All Fields] OR "healthfulness"[All Fields] OR "healths"[All Fields])) OR "wellbeing"[All Fields] OR ("vitalities"[All Fields] OR "vitality"[All Fields]) OR ("social adjustment"[MeSH Terms] OR ("social"[All Fields] AND "adjustment"[All Fields]) OR "social adjustment"[All Fields] OR ("social"[All Fields] AND "functioning"[All Fields]) OR "social functioning"[All Fields]) OR (("cognition"[MeSH Terms] OR "cognition"[All Fields] OR "cognitions"[All Fields] OR "cognitive"[All Fields] OR "cognitively"[All Fields] OR "cognitives"[All Fields]) AND ("functional"[All Fields] OR "functional s"[All Fields] OR "functionalities"[All Fields] OR "functionality"[All Fields] OR "functionalization"[All Fields] OR "functionalizations"[All Fields] OR "functionalize"[All Fields] OR "functionalized"[All Fields] OR "functionalizes"[All Fields] OR "functionalizing"[All Fields] OR "functionally"[All Fields] OR "functionals"[All Fields] OR "functioned"[All Fields] OR "functioning"[All Fields] OR "functionings"[All Fields] OR "functions"[All Fields] OR "physiology"[MeSH Subheading] OR "physiology"[All Fields] OR "function"[All Fields] OR "physiology"[MeSH Terms])) OR ("depressed"[All Fields] OR "depression"[MeSH Terms] OR "depression"[All Fields] OR "depressions"[All Fields] OR "depression s"[All Fields] OR "depressive disorder"[MeSH Terms] OR ("depressive"[All Fields] AND "disorder"[All Fields]) OR "depressive disorder"[All Fields] OR "depressivity"[All Fields] OR "depressive"[All Fields] OR "depressively"[All Fields] OR "depressiveness"[All Fields] OR "depressives"[All Fields]) OR ("anxiety"[MeSH Terms] OR "anxiety"[All Fields] OR "anxieties"[All Fields] OR "anxiety s"[All Fields])) AND ("postpartum period"[MeSH Terms] OR ("postpartum"[All Fields] AND "period"[All Fields]) OR "postpartum period"[All Fields] OR ("postpartum"[All Fields] AND "women"[All Fields]) OR "postpartum women"[All Fields] OR (("postnatal"[All Fields] OR "postnatally"[All Fields]) AND ("womans"[All Fields] OR "women"[MeSH Terms] OR "women"[All Fields] OR "woman"[All Fields] OR "women s"[All Fields] OR "womens"[All Fields])) OR (("breast feeding"[MeSH Terms] OR ("breast"[All Fields] AND "feeding"[All Fields]) OR "breast feeding"[All Fields] OR "breastfeeding"[All Fields] OR "breastfeedings"[All Fields] OR "breastfeeders"[All Fields]) AND ("womans"[All Fields] OR "women"[MeSH Terms] OR "women"[All Fields] OR "woman"[All Fields] OR "women s"[All Fields] OR "womens"[All Fields])) OR (("lactating"[All Fields] OR "lactation"[MeSH Terms] OR "lactation"[All Fields] OR "breast feeding"[MeSH Terms] OR ("breast"[All Fields] AND "feeding"[All Fields]) OR "breast feeding"[All Fields] OR "lactational"[All Fields] OR "lactations"[All Fields] OR "lactators"[All Fields]) AND ("mother s"[All Fields] OR "mothered"[All Fields] OR "mothers"[MeSH Terms] OR "mothers"[All Fields] OR "mother"[All Fields] OR "mothering"[All Fields])) OR ("postpartum period"[MeSH Terms] OR ("postpartum"[All Fields] AND "period"[All Fields]) OR "postpartum period"[All Fields] OR "puerperal"[All Fields] OR "puerperally"[All Fields] OR "puerperant"[All Fields] OR "puerperants"[All Fields]) OR ("maternally"[All Fields] OR "maternities"[All Fields] OR "maternity"[All Fields] OR "mothers"[MeSH Terms] OR "mothers"[All Fields] OR "maternal"[All Fields]))

**Database: Embase <Hinari Access to Research for Health programme> 2020 August 14**

Search query

('anaemia'/exp OR anaemia OR 'anemia'/exp OR anemia OR 'hemoglobin'/exp OR hemoglobin OR 'haemoglobin'/exp OR haemoglobin OR 'hemoglobine'/exp OR hemoglobine OR 'haemoglobine'/exp OR haemoglobine OR 'hb'/exp OR hb OR 'ferritin'/exp OR ferritin OR ferritine) AND ('health related quality of life'/exp OR 'health related quality of life' OR (('health'/exp OR health) AND related AND ('quality'/exp OR quality) AND of AND ('life'/exp OR life)) OR 'quality of life'/exp OR 'quality of life' OR (('quality'/exp OR quality) AND of AND ('life'/exp OR life)) OR hrqol OR 'health status'/exp OR 'health status' OR (('health'/exp OR health) AND status)) AND ('postpartum women'/exp OR 'postpartum women' OR (('postpartum'/exp OR postpartum) AND ('women'/exp OR women)) OR 'puerperal'/exp OR puerperal OR 'breastfeeding women' OR (('breastfeeding'/exp OR breastfeeding) AND ('women'/exp OR women)) OR 'postnatal women' OR (postnatal AND ('women'/exp OR women)))
